# Removal of tumor thrombus from the azygos vein in an esophageal squamous cell carcinoma patient

**DOI:** 10.1186/s13019-020-01092-4

**Published:** 2020-03-26

**Authors:** Qijue Lu, Bowen Shi, Jiang Hong, Hezhong Chen, Chunguang Li

**Affiliations:** Department of Thoracic Surgery, Changhai Hospital, The Second Military Medical University, No. 168 Changhai Road, Shanghai, 200433 China

**Keywords:** Esophageal squamous cell carcinoma, Tumor thrombus, Azygos vein, Neoadjuvant chemotherapy

## Abstract

**Background:**

Esophageal squamous cell carcinoma with tumor thrombus in the arch of the azygos vein has not been reported to date. Neoadjuvant chemotherapy can decrease the stage in patients with advanced preoperative tumor staging, regaining surgical opportunities and significantly prolonging progression-free survival and overall survival. Herein, we present a case of esophageal squamous cell carcinoma accompanied by tumor thrombus in the arch of the azygos vein, and the patient underwent radical surgery after neoadjuvant chemotherapy.

**Case presentation:**

A 63-year-old male with esophageal squamous cell carcinoma was found to have tumor thrombus formation in the arch of the azygos vein. Four courses of neoadjuvant chemotherapy with the TP regimen (paclitaxel plus nedaplatin) were given. Reexamination revealed a significant reduction in tumor and tumor thrombus volume. Therefore, McKeown radical resection for esophageal cancer and removal of the tumor thrombus in the arch of the azygos vein were performed. Postoperative pathology suggested complete remission of the esophageal tumor and the presence of small focal cancer tissues in the arch of the azygos vein.

**Conclusion:**

We report a case of esophageal squamous cell carcinoma with tumor thrombus formation in the azygos vein. We conducted radical resection after 4 rounds of neoadjuvant chemotherapy, and the pathological results revealed complete remission of the tumor. We report our experience addressing this rare case, and we hope to find the underlying mechanism of tumor thrombus formation and whether it has any effects on prognosis in our future study.

## Background

Clinically, there are several studies in the literature regarding cases of renal carcinoma accompanied by inferior vena cava tumor thrombus [1, 2] and liver cancer with portal vein tumor thrombus [3, 4]; however, esophageal squamous cell carcinoma (ESCC) with tumor thrombus in the arch of the azygos vein has not been reported to date. In recent years, neoadjuvant chemotherapy has become increasingly important in the treatment of ESCC; in particular, neoadjuvant chemotherapy decreases the tumor stage in patients with advanced preoperative tumor staging, regaining surgical opportunities and significantly prolonging progression-free survival and overall survival [5]. Herein, we present a case of ESCC accompanied by tumor thrombus in the arch of the azygos vein, and the patient underwent radical surgery after neoadjuvant chemotherapy.

## Case presentation

**Fig. 1:**
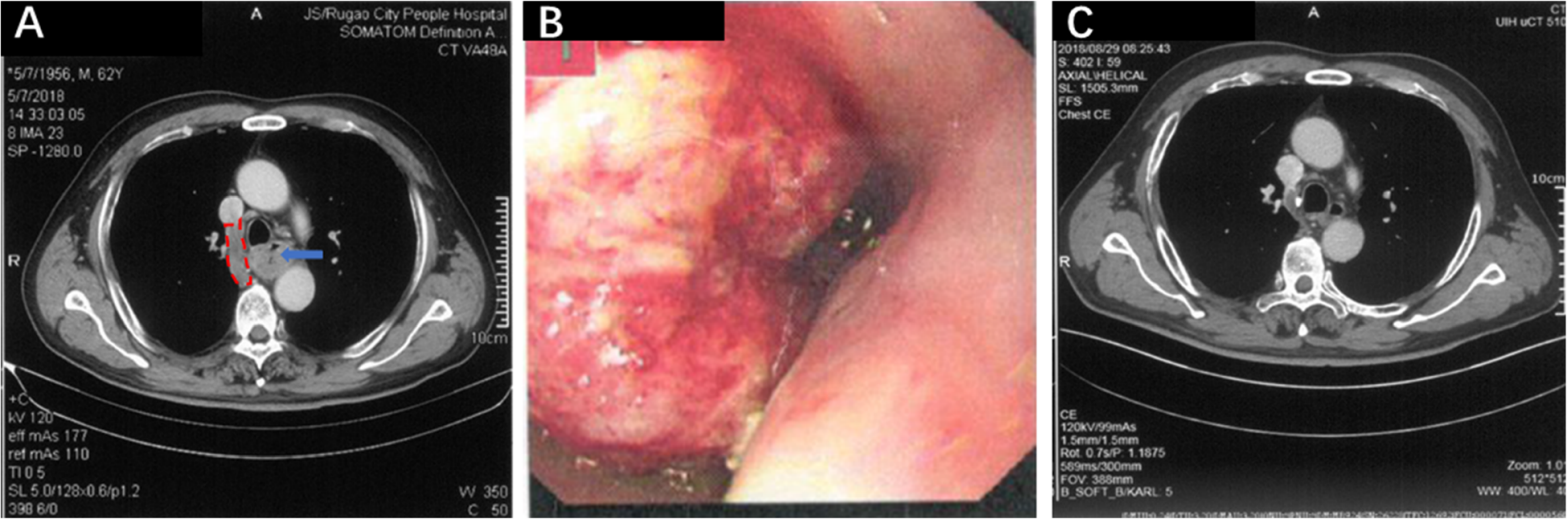
ESCC was diagnosed by CT and gastroscopy. **a** Chest enhanced CT before chemotherapy showed thicken walls of the middle esophagus with tumor thrombus in the arch of azygos vein. **b** Gastroscopic examination showed irregular protuberance in the esophageal lumen, and its surface remained anabrotic, and the tumor basically blocked the esophageal lumen. **c** Chest enhanced CT after neoadjuvant chemotherapy showed a significant reduction in the esophageal tumor and tumor thrombus at the arch of azygos vein

A 63-year-old male with progressive dysphagia for 15 days was found to have thickened walls in the mid-esophageal region and tumor thrombus formation in the arch of the azygos vein (Fig. 1a, S1) according to contrast-enhanced CT; this was accompanied by mediastinal lymphadenectasis. Gastroscopy confirmed the presence of a neoplasm in the esophageal lumen located 28–35 cm away from the incisor. Biopsy suggested poorly differentiated carcinoma of the esophagus, and immunohistochemistry showed a tendency for basaloid squamous cell carcinoma (Fig. 1b). The patient had smoked for 44 years, with 20 cigarettes per day, but had quit smoking for 1 month; the patient had no history of alcohol consumption and denied any history of cardiovascular disease, and no abnormalities were found on the cardiac ultrasound. The patient then received four rounds of neoadjuvant chemotherapy, which consisted of 400 mg Abraxane, 120 mg nedaplatin via intravenous drip on d1, Q3W. Twenty-five days after the last round of chemotherapy, enhanced CT of the chest suggested a significant reduction in the tumor volume, mediastinal lymph nodes, and tumor thrombus in the arch of the azygos vein (Fig. 1c, S2), and general PET-CT showed no distant metastasis.

**Fig. 2:**
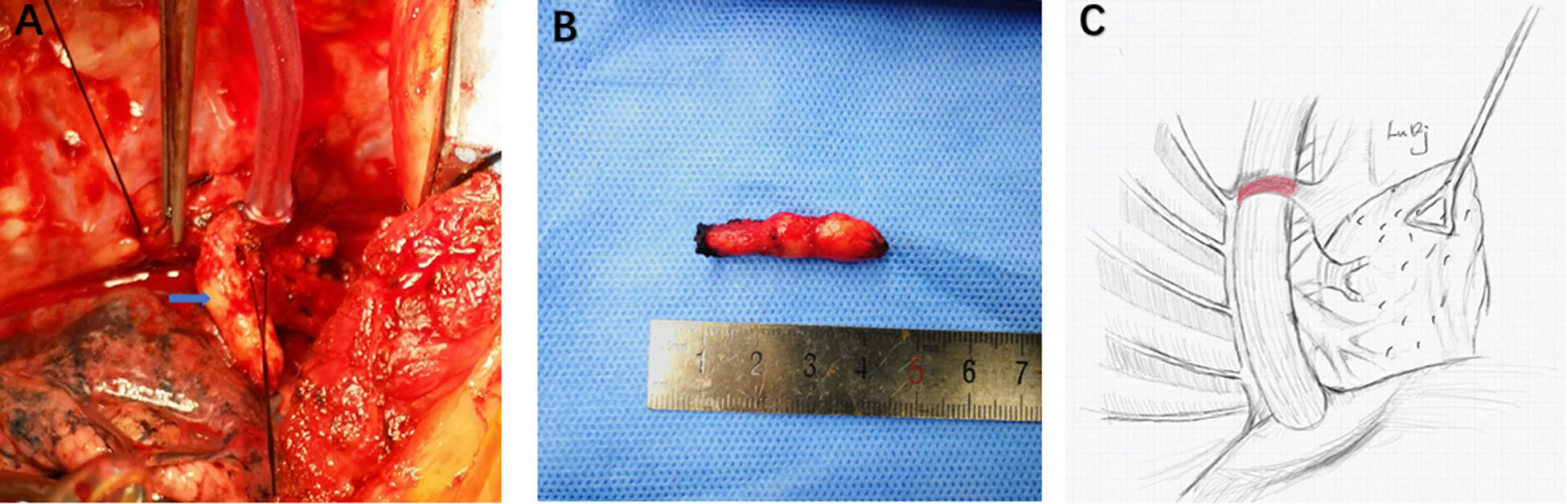
Tumor thrombus at the arch of azygos vein. **a** Intraoperatively, the arch of azygos vein was hard in nature. **b** tumor thrombus was about 3 cm in length. **c** location of the tumor thrombus in the arch of azygos vein, and the red area represents the tumor thrombus

One month later, the patient underwent McKeown radical resection. Intraoperative exploration showed a tumor located at the level of the arch of the azygos vein, measuring approximately 3 cm in length and 3 cm in diameter, with the tumor invading into the esophageal outer membrane. Two enlarged lymph nodes measuring approximately 1 cm in maximum diameter were present around the tumor, and the tumor had a relatively clear boundary with the surrounding tissues. The arch of the azygos vein was hard in nature, wherein the embolus could be palpated (Fig. 2a, b and c). The operative process included entering the posterior-lateral 5th intercostal space of the right chest to expose the esophageal bed and open the mediastinal pleura; the superior vena cava at the arch of the azygos vein was first dissociated, and sidewall forceps were used to clip the converging point of the superior vena cava and azygos vein. This, in turn, prevented tumor thrombus detachment, and then the normal azygous vein was dissociated at the T6 level and blocked. Branches of the intercostal veins were dissociated seriatim and underwent ligation to prevent tumor thrombus detachment countercurrently. Scissors were then used to open the azygos vein, and further exploration showed a clear boundary between the tumor thrombus and the vascular wall of the arch of the azygos vein. There was no invasion or adhesion between the thrombus and the vasculature. After that, blunt and complete dissection of the embolus at the arch of the azygos vein was performed, and the embolus was approximately 1 cm in diameter and 3 cm in length, with no residual embolus observed. Heparin water was used for washing to detach the azygos vein, and then a 5–0 Prolene propene line was used for suturing the azygous vein. We tried to maintain the integrity of the azygos vein during surgery to maintain venous return and to stabilize the gastric tube because of the relative position in the chest. The total length of the esophagus was dissociated. A median incision in the upper abdomen was performed to make a gastric tube, and esophagogastric anastomosis was completed through a right neck incision.

**Fig. 3:**
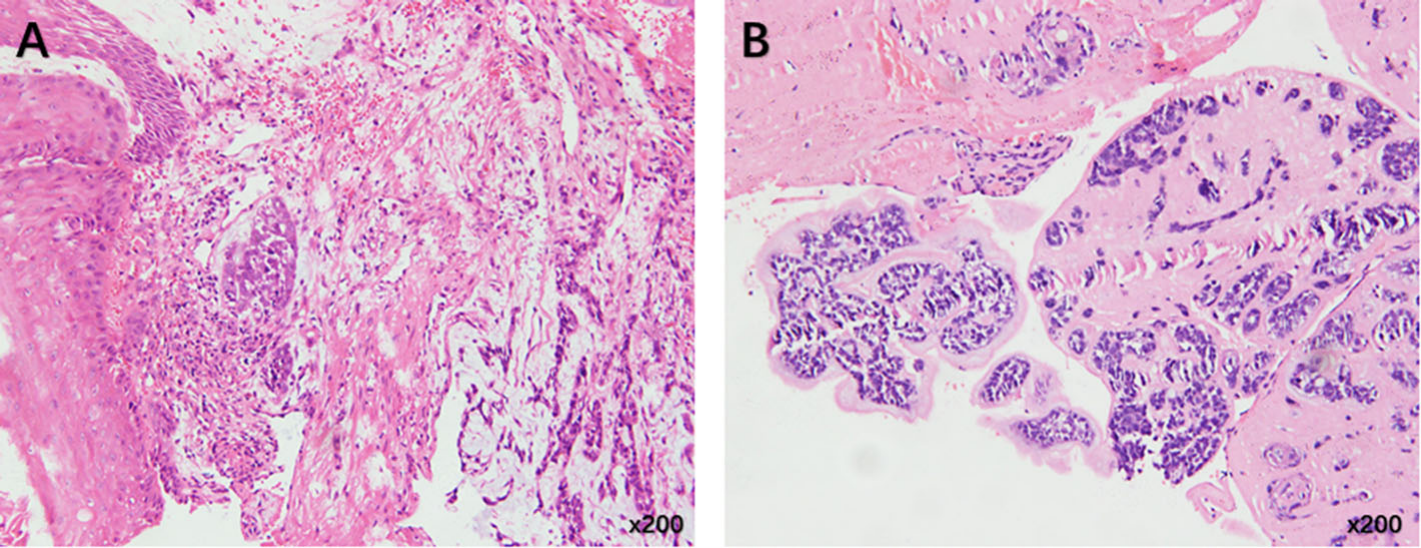
Postoperative pathology. **a** Postoperative pathology suggested complete remission of esophageal tumor. **b** The presence of small focal cancer tissue in the arch of azygos vein

Postoperatively, enteral combined with intravenous nutritional therapy was given. The patient received a clear and liquid diet on day 8 after surgery and was discharged without any severe complications. Postoperative pathology revealed the proliferation of fibrous tissue at the submucosal and muscular layers of the esophageal tumor bed, and a multinucleated giant cell reaction was partially observed, accompanied by infiltration of a small number of lymphocytes and plasmocytes (Fig. 3a). There was no evidence of residual cancer tissue, and complete remission was achieved after neoadjuvant chemotherapy. No metastasis was observed at the mediastinal, celiac, or cervical lymph nodes. Most of the emboli in the azygos vein were necrotic tissues, wherein small focal cancer tissues measuring 3 mm in maximum diameter were present (Fig. 3b).

## Discussion and conclusions

Many studies have mentioned that vascular cancer thrombus is an important factor affecting the lymph node metastasis rate and N stage of esophageal cancer in ESCC [6]; however, the occurrence of esophageal squamous cell carcinoma with tumor thrombus in the arch of the azygos vein or superior vena cava has not been reported to date. To our knowledge, it is of great importance during surgery to prevent detached tumor thrombi from embolizing in the lung and brain. Therefore, the superior vena cava at the arch of the azygos vein was first dissociated, and then sidewall forceps were used for clipping of the converging point of the superior vena cava and azygos vein. Branches of the intercostal veins were dissociated and ligated to avoid tumor thrombus detachment countercurrently. After that, the arch of the azygos vein was cut, and the tumor thrombus was removed successfully. No recurrence or metastasis was observed at 6 months of follow-up, providing a clinical reference for the treatment of similar cases in the future.

The specific mechanism of ESCC with tumor thrombus formation in the azygos vein requires further study. There have been several reports regarding liver cancer with portal vein tumor thrombus. This phenomenon is not only related to the abnormal vascular structure of the tumor, portal vein countercurrent, and blood coagulation function but also associated with the expression of various genes, microRNAs and abnormal proteins. In addition, cytokines may play an important role in the process of cancer cell detachment, attachment, and seeding [7, 8]. Although the tumor in this case was located adjacent to the tumor thrombus of the arch of the azygos vein, its boundary with the arch of the azygos vein remained clear, and direct invasion of the tumor did not cause tumor thrombus formation. Therefore, it was speculated that the mechanism of formation might be related to the combined actions of various genes, the tumor microenvironment and coagulation function. Further accumulation of cases is warranted to determine the mechanism of tumor thrombus formation and whether it has any effects on prognosis.

## Supplementary information

**Additional file 1.** The scan version of original chest CT of the patient before surgery.

**Additional file 2.** The scan version of original chest CT of the patient after 4 rounds of neoadjuvant chemotherapy.

## Data Availability

Not applicable.

## Abbreviations

ESCC: Esophageal squamous cell carcinoma
TP: Paclitaxel plus nedaplatin
CT: Computed tomography
PET-CT: Positron emission tomography-computed tomography
