# Supplementary figures and images for: Removal of tumor thrombus from the azygos vein in an esophageal squamous cell carcinoma patient
Source: J Cardiothorac Surg. 2020 Mar 26;15:52. doi: 10.1186/s13019-020-01092-4 (PMC7104544; doi:10.1186/s13019-020-01092-4)

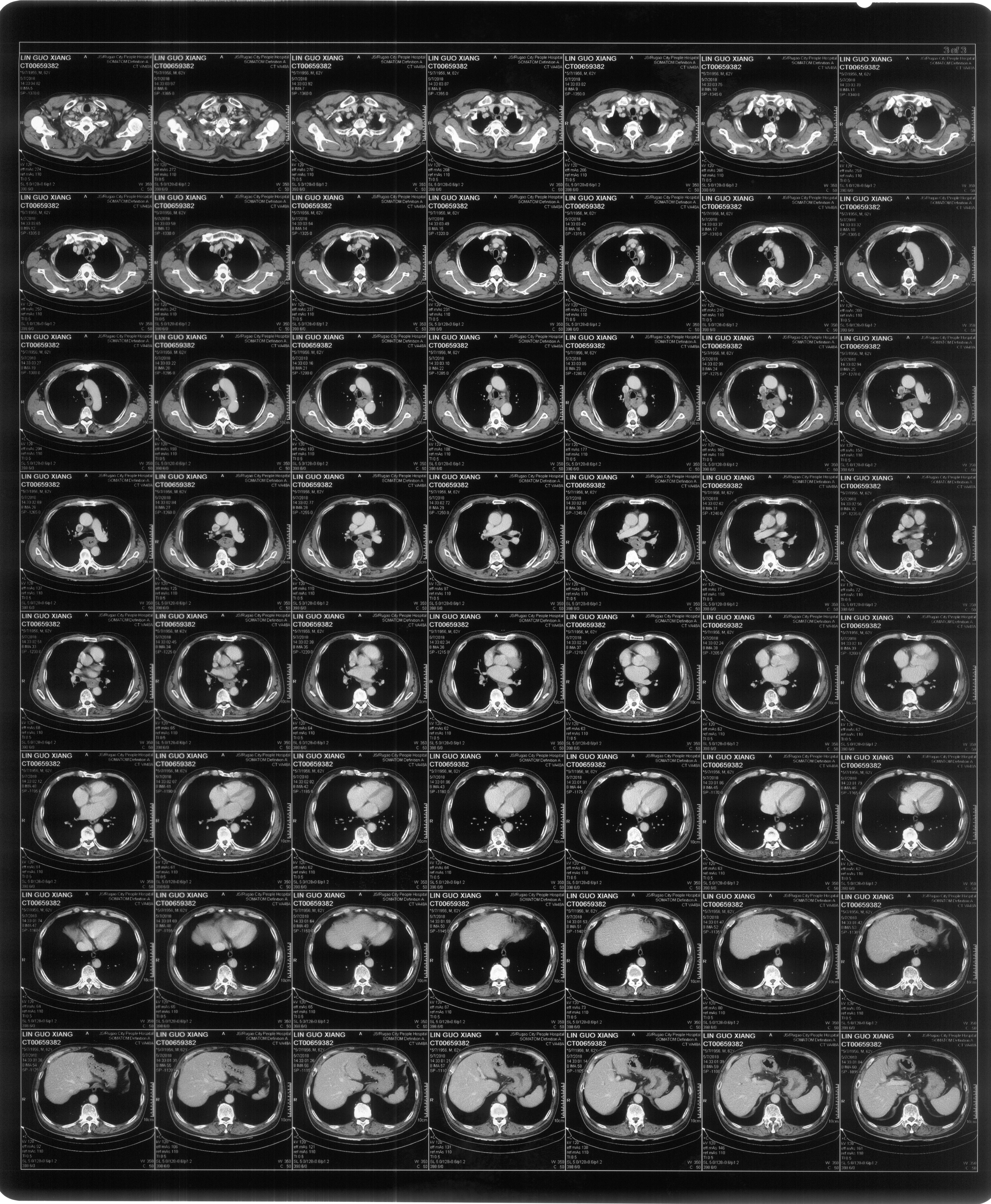

Supplement: Supplementary file 1 — Additional file 1. The scan version of original chest CT of the patient before surgery. [file 13019_2020_1092_MOESM1_ESM.jpg]

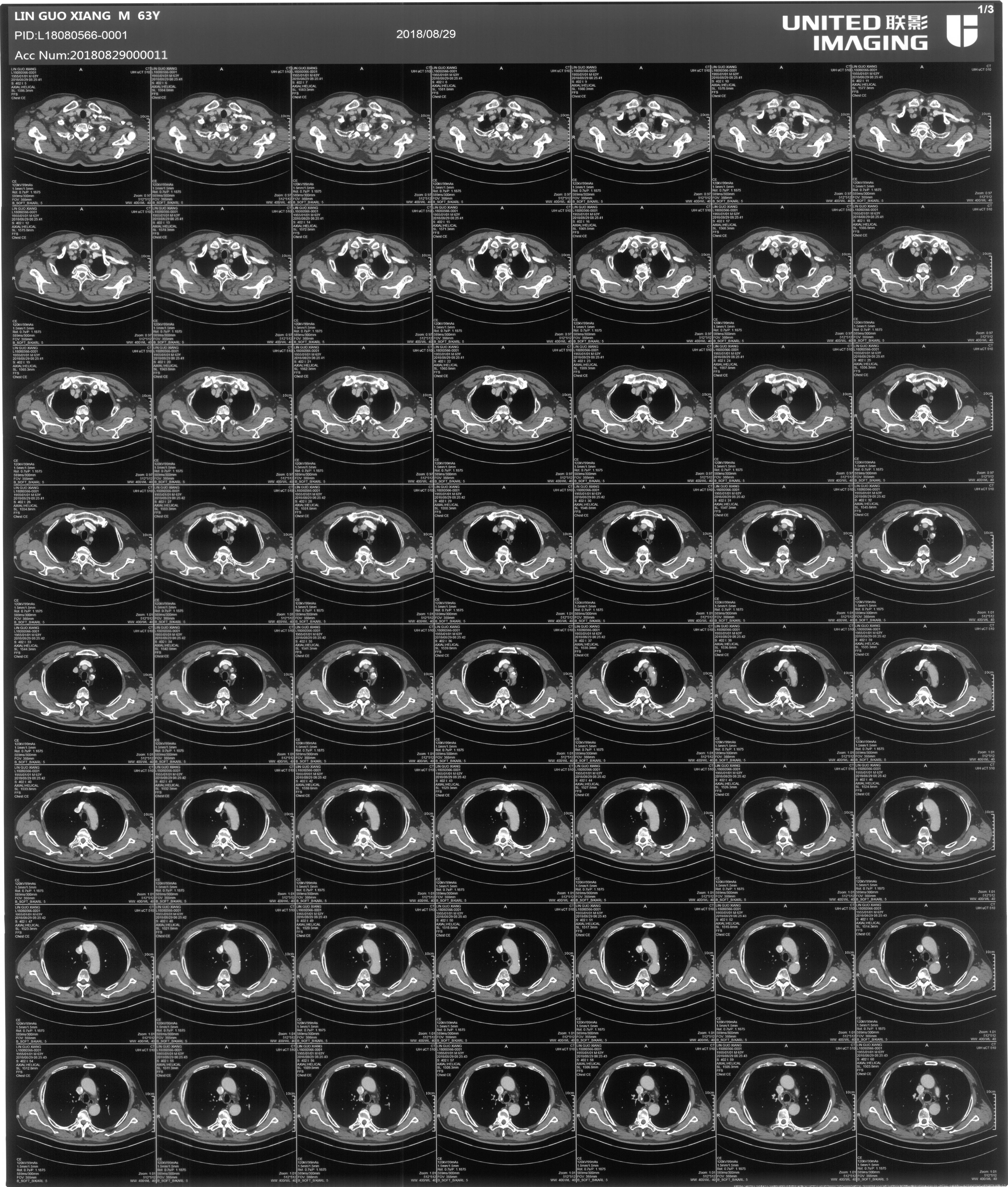

Supplement: Supplementary file 2 — Additional file 2. The scan version of original chest CT of the patient after 4 rounds of neoadjuvant chemotherapy. [file 13019_2020_1092_MOESM2_ESM.jpg]
